# Modified Synthesis and Physicochemical Characterization of a Bioglass-Based Composite for Guided Bone Regeneration

**DOI:** 10.1155/2021/4295433

**Published:** 2021-12-03

**Authors:** Marcos José da Silva, Wellington Alves, Carlos Frederico de Oliveira Graeff, Paulo Henrique Perlatti D'Alpino

**Affiliations:** ^1^Centro Universitário FIEO-UNIFEO, Osasco, SP, Brazil; ^2^Instituto de Pesquisas Energéticas e Nucleares (IPEN), Butantã, SP, Brazil; ^3^São Paulo State University (UNESP), School of Sciences, POSMAT (Post-Graduate Program in Materials Science and Technology), Bauru, SP, Brazil; ^4^Triplet Biotechnology Solutions, São Paulo, SP, Brazil

## Abstract

**Objectives:**

Bioglass composites and polymers are materials of great interest for the medical and dental areas due to their properties, combining the bioactivity of ceramic materials and the mechanical properties of polymers. The purpose of the present study was to develop and to characterize the physicochemical and morphological properties an experimental bioglass-based ternary composite composed associated with sodium carboxymethylcellulose (Na-CMC) and polyvinyl alcohol (PVA). The compatibility of functional groups with bioglass was previously evaluated. The composite was then synthesized and evaluated in terms of morphology, elemental composition, compressive strength, porosity, and bioactivity.

**Materials and Methods:**

The bioglass was previously synthesized using a sol-gel route and characterized using FTIR analysis to identify the functional groups. The bone graft composite was then synthesized associating the bioglass with PVA, surfactant Triton *X*, and Na-CMC. The composite was then morphologically characterized using SEM/EDS. The porosity of the composite was analyzed using µCT, which also provided the composite compression strength. The composite was then evaluated in terms of its bioactivity using SEM/EDS analyses after immersion in SBF for 12, 24, 48, and 72 h.

**Results:**

FTIR analysis confirmed, among other components, the presence of Si–O–Ca and Si–O–Si bonds, compatible with bioglass. SEM analysis exhibited a composite with a porous structure without spikes. The elemental mapping confirmed the presence of Si, Ca, and P in the composite. µCT analysis demonstrated a porous structure with 42.67% of open pores and an average compression strength of 124.7 MPa. It has also demonstrated ionic changes in the composite surface after immersion in SBF, with increasing detection of Ca and P as a function of time, highlighting its chemical bioactivity.

**Conclusions:**

It can be concluded that the proposed bioglass-based composite presents a three-dimensional, well-structured, chemically bioactive porous structure, mechanically resistant for being reinforced with polymeric phases, with promising results as a synthetic bone graft, which makes it suitable for guided bone regeneration.

## 1. Introduction

The main problem reported in the literature in the application of pure hydroxyapatite as a bone graft, regardless of its origin, is the fact that it is an inert material, not bioactive [1]. Hydroxyapatite has a “structural” role, configuring a scaffold where cell deposition and proliferation with osteoblastic activity occur, whose source of osteoprogenitor cells is provided by the peripheral bone tissue [2]. Hydroxyapatite, although biocompatible, has osteoconductive activity and not properly osteoinductive activity [3, 4]. Osteoconductive activity does not stimulate bone neoformation, and the bone grafts remain unchanged, encapsulated, or reabsorbed [3]. This directly affects the healing time postop for many months [5].

Despite the technology used in the development of bone grafts of animal origin, especially bovine, it is observed that there are problems in quality control, reflecting the lack of standardization between batches that have different physical properties (crystallinity, porosity, among others) [6]. These characteristics have a direct impact on the biological response, with reflexes on the regenerative capacity, and different clinical results [7]. In addition, no embedded innovation is observed in these products considering that bovine bone is simply processed, sterilized, and packaged. Thus, there is a need for innovative biotechnological products that present osteoinductive activity, an important missing characteristic in the commercial products desired by clinicians. In addition, quality products offer greater safety for clinicians and patients, reducing postoperative complications, accelerating the healing process, with less impact for patients [8, 9].

Numerous studies aiming at the production of porous bioceramics have been developed, including the incorporation of additives to increase the porosity of the material to enable its osteointegration [1, 10]. Among them, bioglass is widely used in tissue engineering in the form of matrices for bone regeneration [11], which cause a specific biological response at the material interface, resulting in the formation of a connection between the tissue and the implanted material [12]. Concerns have been expressed over the porosity and brittleness of the bioactive glass, which makes it not suitable for bone graft substitute [13]. In spite of these flaws, bioglass can still be used to enhance the efficacy of existing bone substitute materials. In this way, the present study proposed the development of this experimental bioglass-based composite for guided bone regeneration. The present study aimed to develop and characterize an experimental bone graft based on a ternary composite composed of bioglass, sodium carboxymethylcellulose (Na-CMC), and polyvinyl alcohol (PVA). For that, the synthesis and physicochemical characterizations of the experimental composite were described.

## 2. Materials and Methods

### 2.1. Bioglass and Composite Synthesis

Bioactive glasses were synthesized using a modified route, according to a previously published study [14]. The bioglass was synthesized by means of the sol-gel route alkoxide method, using PA reagents. The precursors of SiO_2_ and P_2_O_5_ used were tetraethyl orthosilicate (TEOS, Aldrich Chemical Company, Inc.) and triethyl phosphate (TEP, Aldrich Chemical Company, Inc.), respectively. Calcium nitrate (Ca(NO_3_)_2_).4 H_2_O was used as a precursor of CaO. The hydrolysis was catalyzed by nitric acid (HNO_3_) and water was obtained by the process of reverse osmosis. The samples were then sintered at 750°C for 260 minutes and the resulting solid was ground in a ball mill until micrometric powders were obtained. The PVA Mowiol 18–88 (Sigma Aldrich), Na-CMC (Sigma Aldrich), and the surfactant Triton X-100 (Sigma Aldrich) were used to obtain the composite. After obtaining the micrometric granules of bioglass, the composite was then produced.

In a beaker containing 100 mL of water obtained by reverse osmosis, under stirring and heating at 60°C, 5% w/v of PVA was solubilized. The solution was kept under stirring for 40 minutes until the complete dissolution of the PVA. Heating was removed and stirring was continued until reaching room temperature. Then, 3% v/v of the surfactant Triton *X* was added to the solution and stirring was continued for another 20 minutes. Then 3% w/v Na-CMC was added to the solution and stirring was continued for another 40 minutes. Then, 50% w/v of micrometric granules of bioactive glass were gradually added to the solution in a propeller agitator and the composite remained under stirring for another 20 minutes and then poured into containers with varying shapes and volumes. The containers containing the composites were placed in an oven at 37°C for 72 h. In the present study, the concentrations of PVA and Na-CMC were 5% w/v and 3% w/v, respectively.

### 2.2. Bioglass Characterization Using Fourier Transform Infrared (FTIR) Analysis

The identification of functional groups in the synthesized bioglass was performed using a spectroscopic technique (Fourier transform infrared (FTIR), Thermo Nicolet Nexus 4700 FTIR Spectrometer, Ramsey, MN, USA), with the wavelength ranging from 4000 to 400 cm^−1^. Spectra were obtained by placing the materials directly on the diamond crystal localized at the ATR attachment. Infrared spectra of the polymerized products were obtained using 16 scans at a resolution of 4 cm^−1^. Three replications were performed.

### 2.3. Morphological Characteristics of the Composite by Scanning Electron Microscopy (SEM), Imaging Observation, and Energy-Dispersive X-Ray Spectroscopy (EDS)

The specimens of the composite were sputter-coated (40 mA for 120 s) with gold/palladium (SCD 050; Balzers, Schaan, Liechtenstein) to characterize the inorganic phase of the composite by means of SEM (JSM 5600LV - JEOL, Tokyo, Japan) under the secondary electron mode (*n* = 3). Before the SEM analysis, the specimens were dehydrated in silica gel for 24 h and then submitted to carbon evaporation (SCD 050, Balzers, Schaan, Liechtenstein) for elemental analysis using EDS under a backscattered electron mode operating in high vacuum mode and an accelerating voltage of 15 kV. Representative images of selected areas of the sputter-coated specimens were taken to characterize the morphological aspect of the composite, while a qualitative elemental analysis was performed on the carbon-evaporated ones.

### 2.4. Microcomputed Tomography (µCT) Analysis

The specimens were mounted on stubs fitting the specimen stage of a high resolution Skyscan Bruker 1172 Microtomography (DKSH Korea Ltd., Seoul, South Korea). The pixel size was 9.28 *μ*m and the total image size had 2000 × 1336 pixels. The specimens were scanned at a voltage of 89 kV and the applied current of 112 µA. The exposure time was 270 ms per image and the total acquisition time lasted 16 min for each sample. After scanning, the image dataset was reconstructed into tomographic sections by the NRecon software used for volumetric analysis and to create 3D models. The percentage of total porous, open pores, and closed pores in the specimens were calculated. The average and standard deviation of the pores were then calculated (*N* = 3).

### 2.5. Composite Compression Strength

A compression test was also performed using a specific µCT accessory for testing materials. Three specimens of the composite with dimensions 0.5 × 0.5 × 0.8 cm were tested. The compression load was applied in the major axis of the composite block using a specific MicroCT accessory for material testing. The loading was carried out at a speed of 0.03 mm/min and at a room temperature of 22 °C and the environmental humidity was kept below 10% relative humidity [15, 16]. The test was conducted at a constant displacement from the base of the accessory and ended at the maximum load promoted by the device, that is, 22 kg. After that, the pressure was relieved at the same rate of displacement from the base of the accessory. The data demonstrating the mechanical behavior of the composite under load (in Newtons) was plotted *vs.* the distance (in mm). The average compression strength was calculated in MPa.

### 2.6. In Vitro Composite Bioactivity Assessment Using SEM/EDS

The specimens were immersed in a container containing a solution of Simulated Body Fluid (SBF). The containers were placed in a water bath with controlled temperature and kept at 37°C. After different evaluation times (12, 24, 48, and 72 h), the specimens were removed, dried, and analyzed using SEM (TESCAN Scanning Electron Microscope, Vega 3 XMU) using a low vacuum (500 Pa), equipped with a Tungsten filament and Energy-Dispersive Spectroscopy (EDS) detector. The images were obtained in secondary electron mode. Before the SEM analysis, specimens were dehydrated in silica gel for 24 h and then sputter-coated with gold, as previously described.

## 3. Results

**Figure 1:**
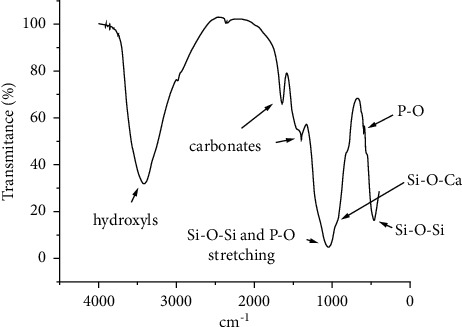
FTIR spectrum of synthesized bioglass.

Figure 1 shows the FTIR spectrum obtained for the synthesized bioglass. FTIR analysis demonstrated functional groups compatible with bioglass. It is possible to observe a peak at 465 cm^−1^, which refers to Si–O–Si folding connections. At 601 cm^−1^, the peak refers to P–O vibrational folding mode of the tetrahedral PO_4_^−3^ in the crystalline carbonated apatite, possibly due to the reaction of the bioglass powder with atmospheric humidity. The inclination around 930 cm^−1^ is associated with the Si–O–Ca vibration mode. The region around 1045 cm^−1^ refers to the asymmetric stretching of Si–O–Si and the vibration of stretching of P–O. Bands referring to carbonates are present at 1395 and 1647 cm^−1^ and bands referring to hydroxyls at 3410 cm^−1^ [17, 18]. The peaks shown are compatible with the composition of a bioglass with a mass of 58% Si presented in the literature [14].  Table 1 lists the infrared frequencies and band assignments of the composite characterized according to the previously described.

**Figure 2:**
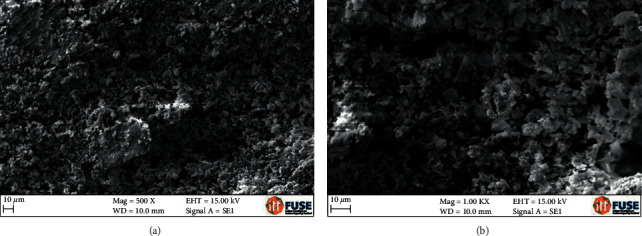
Representative SEM photomicrographs of the composite exhibiting a porous structure and absence of spicules, as well as pore size variations ((a) 500X magnification and (b) 1000X magnification).

**Figure 3:**
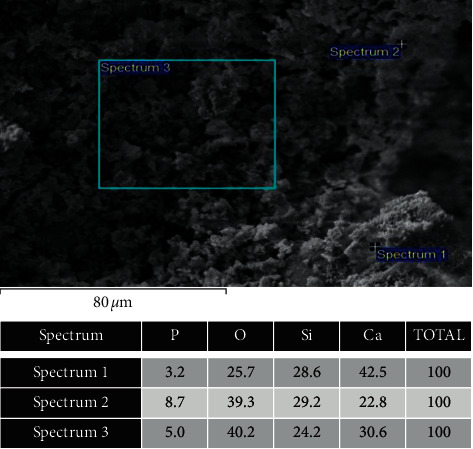
Representative scanning electron micrographs of the composite and the elemental mapping at different areas. EDS detected stronger silicon signals of Ca, Si, P, and O.

Figures 2(a) and 2(b) show representative scanning electron micrographs of the composite at different magnifications, exhibiting a porous structure with absence of spikes. It can be observed that the composite has apparent open pores, which is an important prerequisite in guided bone regeneration. In addition, it was clearly demonstrated that the pore geometry and size are variable, but they are evenly distributed in the specimen. The elemental mapping analysis of the composite confirmed the presence of Si, Ca, and P (Figure 3).

**Figure 4:**
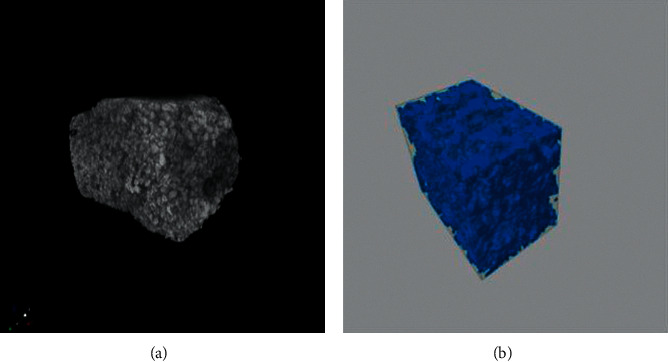
Porosity analysis of the composite using µCT analysis of the porosity of the composite (a). 3D models were created and a volumetric analysis of the porous was performed (b).

Figures 4(a) and 4(b) exhibit the representative images of the tomographic sections of the bone graft composite. The determined average of total pores was 42.78% (±0.33), with 42.67% (±0.43) of open pores and 0.02% (±0.01) of closed pores. A high porosity and interconnectivity were also observed between the pores, in addition to structural organization and homogeneous distribution of the pores in the composite.

**Figure 5:**
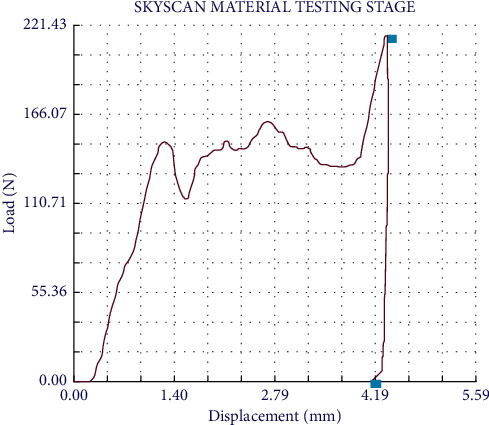
Mechanical behavior of the composite under load (in Newtons) plotted vs. distance (in mm).

Figure 5 demonstrates the mechanical behavior of the composite under load by plotting the force *vs.* distance. The curve shows that the elastic regime of the composite corresponds to the line between 0 and 1.3 mm, exhibiting an elastic deformation in this interval. From this point on, changes in the curve occurred due to the porosity of the composite, which are starting points, and/or sites of propagation of small cracks. Although the presence of polymers allows for greater plastic deformation, the material tends to “collapse” at the end of the test. After the loading reached the peak, the stress fell immediately and abruptly, without residual strength after the rupture of the specimens. The specimens showed an average compressive strength of 124.7 (±1.4) MPa.

**Figure 6:**
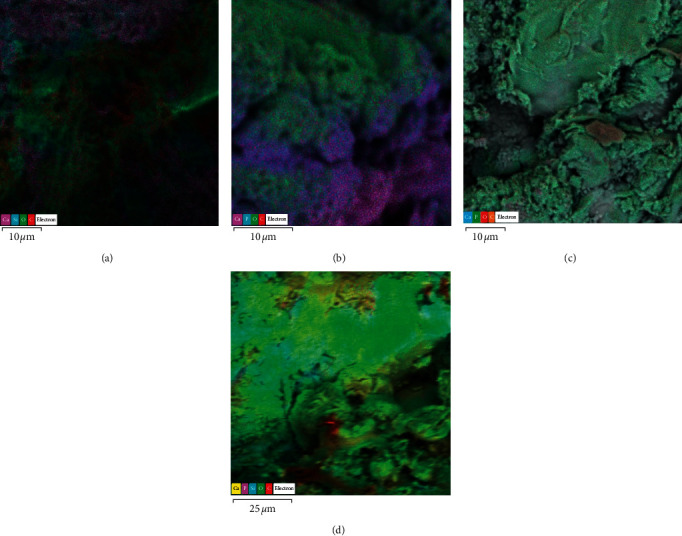
Elemental mapping showing the elemental distribution in the composite after immersion in SBF for 12 (a), 24 (b), 48 (c), and 72 h (d). The amounts of sodium, phosphorus, and calcium varied as a function of the evaluation time.

**Figure 7:**
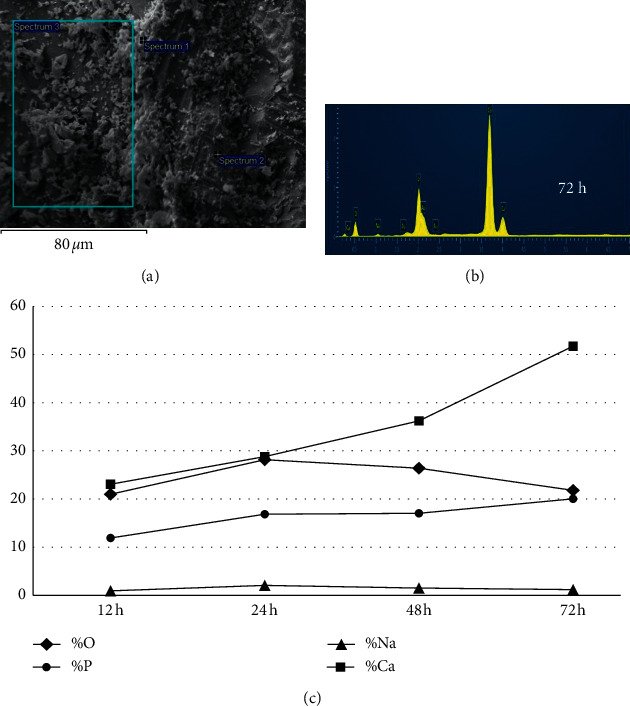
(a) Representative images by SEM highlighting the 3 different areas analyzed. (b) Representative spectrum obtained by EDS from a specimen immersed in SBF. (c) Elemental distribution by MEV/EDS as a function of time.

Figure 6 exhibits the elemental distribution of Si, Ca, P, and Na in the composite after immersion in SBF after 12, 24, 48, and 72 h evaluated by SEM/EDS. The amounts of sodium, oxygen, phosphorus, and calcium varied as a function of the evaluation time, according to the color-coded legends.  Figure 7(a) exhibits a representative scanning electron micrograph highlighting 3 different areas analyzed after immersion in SBF. An ascending detection of Ca and P as a function of time was clearly demonstrated (Figure 7(b)), confirming the bioactivity of the bone graft composite.

## 4. Discussion

Synthetic or natural materials presenting proved cellular interactions, bioabsorption, and biocompatibility and improved mechanical properties have been used in the medical and dental areas [19]. To obtain greater similarity of scaffolds with bone tissue, polymer-ceramic combination appears as an excellent alternative, due to the fact that bone tissue is a complex combination of polymer (collagen) and ceramic (hydroxyapatite) [20]. A recent study demonstrated that the combination of polymers and ceramics is used for the manufacture of scaffolds for tissue engineering [21]. Ceramic materials such as hydroxyapatite, tricalcium phosphate (TCP), calcium silicate, and bioglass ceramics are commonly used in this application [21]. A porous composite to be applied as a scaffold in tissue engineering needs to encompass important characteristics such as biocompatibility, osteoconductivity, interconnected porous structure, appropriate mechanical strength, and biodegradability [22]. Although the characteristics of porosity and brittleness make the bioactive glass impeditive to be a suitable bone graft substitute alone, it has been applied to enhance the efficacy of existing bone substitute materials [23]. It has been reported that a damaged bone tends to quickly recover its original strength when repaired using a combination of a composite and bioactive glass when compared to bone repair using a composite alone [13]. Bioactive polymer composites can be designed to mimic the behavior of bioactive glasses regarding in *vitro* bioactivity, by favoring the release of silicate and phosphate ions when exposed to body fluids [24].

According to the results (Figure 1), the functional groups obtained in the FTIR analysis were compatible with bioglass [25, 26]. The presence of Si, Ca, and P was also confirmed through the analysis using MEV/EDS (Figure 3). It was possible to observe the morphology of the internal and external structure of the scaffolds and to evaluate the shape and opening of the pores of this structure. As observed in Figures 2(a) and 2(b)), the composite exhibits apparently open pores in different areas of the same specimen, which is one of the prerequisites for its use in bone regeneration [27]. In addition, the geometry and size of these pores were found to be variable but evenly distributed. The morphology of the sintered porous composite is the result of the process of adding polymers to reinforce it, such as PVA and Na-CMC, and a porogenic agent, such as Triton *X*, to improve its porosity during its synthesis and subsequent drying process to provide a porous morphological characteristic for an application as a bone substitute.

The structure of scaffolds is in general complex, not easily interpreted in terms of pore shape and size, especially in a 3D analysis. In this manner, characterizations using different techniques are usually required for this purpose. As previously pointed out, a well-structured and organized composite was obtained, with a homogeneous distribution of interconnected pores in the composite with a percentage of 42.67% of interconnected pores (open pores) and only 0.02% of closed pores. Until the fracture point, slightly noticeable plastic deformation stages can be observed, with a clear yield point (Figure 5). The porous structure of the composite helps to explain the composite behavior in the compressive strength test, with an average end compressive strength of 124.7 MPa. Conversely, it has been claimed that the porosity in composite materials correlates with the plastic deformation due to the formation and propagation of cracks in the critical points [28, 29]. When developing a synthetic bone graft, the search for balanced formulae to provide a porous, well-structured scaffold with improved mechanical strength is of paramount importance.

According to a previous study [30], the pore size should present a balance between obtaining optimal cell attachment and facilitating bone growth. Mean pore size is an essential aspect of scaffolds for tissue engineering. If the pores are too small, cells are not able to migrate in towards the center of the construct, thus limiting the diffusion of nutrients and the removal of waste products. Conversely, if the pores are too large, there is a decrease in the specific surface area available, limiting cell attachment. Up to now, this relationship between pore size and cell activity in a bone graft is not completely understood as previous studies in bone tissue engineering have indicated a range of mean pore sizes (96–150 µm) to facilitate optimal attachment. Other studies pointed out that, for a successful bone growth in scaffolds, larger pores (300–800 µm) would be important. The optimization of the scaffold microstructure in terms of porosity, mean size, and size distribution of pores and pores interconnectivity is a complex task [31].

Various compounds have been studied aiming at producing scaffolds for tissue engineering, which include natural polymers, such as collagen, gelatin, and chitosan, and also synthetic polymers that include polyglycolic acid (PGA) and its copolymers (PLGA) [32]. Among the natural polymers derived from glucose, cellulose and its derivatives have attracted considerable attention for applications in the biomedical field due to their biocompatible polymers and suitable physical and mechanical properties [33]. CMC is an anionic polymer derived from cellulose, which creates a transparent gel when dissolved in water. Cellulose-based gels are also biocompatible, biodegradable, and transparent, with low cost, making CMC suitable for many applications in tissue engineering [34]. CMC molecules are usually shorter than those that they originate (cellulose), with areas of greater and lesser substitution, which will result in different viscosities [35].

Purified grade Na-CMC, a widely used derivative of cellulose, is a powder with a color that varies from white to yellowish, hygroscopic, and free from agglomeration, with a wide viscosity range and excellent solubility in cold or hot water due to the presence of substituents in the cellulose chain, which facilitate water permeation [36]. The presence of Na-CMC in a cellulose gel base polyelectrolyte anchored in the network, which shows sensitivity to pH and ionic strength variations [35]. Blending of different polymers is an extremely attractive, inexpensive, and advantageous method to obtain a novel structured composite polymer [34, 37]. Among other polymers, PVA is a good candidate for the preparation of hydrogels which can be cross-linked by using several methods, which includes physically thermal cycling [37].

PVA is a hydrophilic polymer with excellent biocompatibility and has been applied in for tissue engineering applications due to its favorable properties such as hydrophilic nature, biodegradability, excellent biocompatibility, and suitable mechanical strength [38]. To explore their applicability in potential research fields, other polymerization procedures such as copolymerization have also been applied for PVA-based biomaterials [39]. PVA has been also used in the manufacture of composites due to specific characteristics, such as easy solubilization in water-alcohol mixtures used in the sol-gel method and it also favors the production of homogeneous materials with a wide range of compositions [40]. In a previous study, a composite associated PVA and bioglass were synthesized by an emulsion freeze-drying process to obtain a porous 3D scaffold [41]. The composite scaffold was biomimetic and bioactive, also favoring the mineralization process by forming a hydroxycarbonate apatite layer, when immersed in simulated body fluid for a 14-day period. As PVA is part of the formulation of the experimental bone graft, the composite should be applied in the surgical site, and it would be expected to be invaded by the blood tissue and by a population of odontoblastic cells. In this manner, once the bone graft is osseointegrated, there are no concerns regarding PVA accumulation in living organisms considering that it can be either locally incorporated or eliminated from the surgical site.

When a bioglass is exposed to an aqueous environment, there is a localized breakdown of the silica network due to the loss of sodium [26]. This leads to the formation of Si(OH)_4_ groups, which tends to repolymerize into the silica-rich surface layer. After the formation of this silica-rich layer, an amorphous calcium phosphate layer tends to form on the glass surface, which allows the incorporation of biological moieties on its surface, such as blood proteins, growth factors, and collagen [26]. Simultaneously, the Ca and P released from the glass surface tend to form a calcium phosphate layer which may crystallize as a hydroxycarbonate apatite layer, regarded as the bonding layer [11].

It has been previously reported that the release of ionic components from the glass surface seems to continue for long periods, enhancing the development of surface reactive layers [11]. This sequence of chemical events confirms the bioactivity of the bioglass due to its characteristic “bonding to bone” [42]. In the present study, the *in vitro* bioactivity analysis of the composite demonstrated the distribution of the elements Si, Ca, and P in different areas (Figure 6), with ascending concentrations of Ca and P as a function of the evaluation time, which leads to the formation of an amorphous calcium phosphate layer on the composite surface, confirming the bioactivity of the composite (Figure 7). With this composition, the proposed bioglass-based composite was synthesized and characterized demonstrating important physicochemical properties, such as bioactivity, mechanical properties compatible with bone, and morphological characteristics, such as porosity and uniform pore distribution that makes it suitable for bone substitute.

## 5. Conclusions

In the present study, we aimed to develop a ternary matrix composite consisting of bioglass/Na-CMC/PVA. Taken together, the results allow us to conclude that a three-dimensional well-structured, chemically bioactive porous composite was obtained, mechanically resistant for being reinforced with polymeric phases. Further *in vivo* and *in vitro* studies are necessary to demonstrate the ability of the composite to facilitate the population of odontoblastic cells and to support mechanical stresses in a clinically relevant scenario to indicate it as a synthetic bone graft.

## Figures and Tables

**Table 1:** Infrared frequencies and band assignments of the bone graft composite.

Vibrational mode	Attribution	Wavenumber (cm^−1^)
Bending	Si-O-Si	465
Symmetric stretch	P-O bond at PO43-	601
Stretching	Si-O-Ca	930
Asymmetric stretch	Si-O-Si; P-O	1045
Stretching	C-O-C/C-O bonds (carbonates)	1647-1395
Stretching	Groups O-H	3410

N=3.

## Data Availability

The data used to support the findings of this study are included within the article.
